# A Pilot Test of Self-Affirmations to Promote Smoking Cessation in a National Smoking Cessation Text Messaging Program

**DOI:** 10.2196/mhealth.5635

**Published:** 2016-06-08

**Authors:** Jennifer M Taber, William M.P Klein, Rebecca A Ferrer, Erik Augustson, Heather Patrick

**Affiliations:** ^1^ Behavioral Research Program National Cancer Institute National Institutes of Health Rockville, MD United States; ^2^ Basic Biobehavioral and Psychological Sciences Branch Behavioral Research Program, National Cancer Institute National Institutes of Health Rockville, MD United States; ^3^ Applied Behavior Change Science Envolve PeopleCare Bethesda, MD United States

**Keywords:** self-affirmation, smoking cessation, mHealth, text messaging, theoretical study, self concept, motivation

## Abstract

**Background:**

Although effective smoking cessation treatments, including mHealth interventions, have been empirically validated and are widely available, smoking relapse is likely. Self-affirmation, a process through which individuals focus on their strengths and behaviors, has been shown to reduce negative effects of self-threats and to promote engagement in healthier behavior.

**Objective:**

To assess the feasibility of incorporating self-affirmations into an existing text messaging-based smoking cessation program (Smokefree TXT) and to determine whether self-affirmation led to greater engagement and higher cessation rates than the standard intervention.

**Methods:**

Data were collected from smokers (*n*=1261) who subscribed to a free smoking cessation program and met eligibility criteria. The intervention lasted 42 days. The original design was a 2 (Baseline affirmation: 5-item questionnaire present vs absent) × 2 (Integrated affirmation: texts present vs absent) factorial design. Only 17 eligible users completed all *baseline affirmation* questions and these conditions did not influence any outcomes, so we collapsed across baseline affirmation conditions in analysis. In the *integrated affirmation* conditions, affirmations replaced approximately 20% of texts delivering motivational content.

**Results:**

In all, 687 users remained enrolled throughout the 42-day intervention and 81 reported smoking status at day 42. Among initiators (*n*=1261), self-affirmation did not significantly improve (1) intervention completion, (2) days enrolled, (3) 1-week smoking status, or (4) 6-week smoking status (all *P*s>.10); and among the 687 completers, there were no significant effects of affirmation on cessation (*P*s>.25). However, among the 81 responders, those who received affirmations were more likely to report cessation at 6 weeks (97.5%; 39 of 40) than those not given affirmations (78.1%; 32 of 41; *χ*^2^(1)=7.08, *P*=.008).

**Conclusion:**

This proof-of-concept study provides preliminary evidence that self-affirmation can be integrated into existing text-based cessation programs, as the affirmations did not lead to any adverse effects (ie, less engagement or lower rates of cessation). Among those who reported smoking status at the end of the intervention period (6.4% of eligible respondents), affirmations facilitated cessation. This study provides a “proof-of-concept” that brief, low-touch interventions may be integrated into a text messaging program with potential benefits, minimal disruption to the program or users, and little cost. Many questions remain regarding how self-affirmation and similar approaches can promote engagement in population interventions.

## Introduction

Effective smoking cessation treatments, including those delivered via mobile health platforms, have been empirically validated and are widely available [1-3]. However, quitting smoking is challenging and relapse is likely [4]. Smokers may feel threatened by temptation to smoke if they perceive an inability to meet cessation challenges. Self-affirmation [5]—or focusing on strengths and values—can offset self-threats and promote healthier behaviors [6-8]. In prior research, self-affirmed smokers took more antismoking brochures and reported greater self-efficacy to quit and higher intentions to reduce smoking [9-10]. When combined with other intervention strategies, self-affirmed undergraduates actually smoked less [11]. Smokers who self-affirm may be more successful at quitting, because temptation may be seen as less threatening to competence or self-control.

Self-affirmation interventions may be scalable and disseminable, as demonstrated elsewhere (eg, education [12]), and given that lasting effects emerge with minimal interventions [13,14]. Self-affirmation operates through recursive, self-perpetuating processes, by motivating individuals to capitalize on preexisting resources to facilitate change [13]. Implementing brief affirmations into at-scale cessation interventions may increase their effectiveness. This study assessed the feasibility of incorporating self-affirmations into an existing text messaging-based smoking cessation program and examined whether self-affirmation might facilitate greater engagement and higher cessation rates than a standard intervention.

## Methods

### Procedure

Data were collected from smokers who subscribed to a free smoking cessation program, implemented by the US National Cancer Institute (Smokefree TXT; [15]), online or through text. Smokefree TXT (launched in 2009) was designed following a review of text-based cessation programs and input from cessation and mHealth experts. The library has been updated multiple times following user testing and analysis of user data. Taxonomic analysis indicates that the text program provides 35 different behavioral change techniques [16].

Upon enrollment, users of the program reported their mobile number, age, gender, state of residence, and smoking frequency, and were instructed to set a quit date between today and 14 days in the future. Users were informed that the program provided “24/7 encouragement, advice, and tips to help smokers quit smoking and stay quit.” As part of the standard program, users received daily text messages designed to improve self-efficacy to quit and to provide motivation. Texts were sent up to 14 days before users’ self-selected quit day and during the 42-day period following users’ self-selected quit day. Before the quit day, all users received 2-3 texts/day (with the exception of the baseline affirmation prior to enrollment, which consisted of 5 texts). For the first two weeks after quit day, users received 3-4 texts/day. From day 15 to 42, users received 1-3 texts/day. The first text of the day was sent at 9am, followed by 12pm, 3pm, and 6pm, for as many texts as were sent that day. All texts were unique and the order was standardized within each condition. Users could disenroll anytime. With the exception of the self-affirmations, no changes were made to the existing program to modify it for research use. Thus, these data represent an evaluation of incorporating self-affirmation into a readily available, already disseminated intervention.

Users who subscribed during a 6-week period in fall 2014 were automatically enrolled into one of three enhanced intervention conditions based on which day they enrolled. Enrollment into the three conditions was distributed across days of the week and throughout the duration of this 6-week period. Control data were drawn from users who subscribed during 2 weeks in September immediately prior to this 6-week period. The original design was a 2 (Baseline affirmation: 5-item kindness questionnaire present vs absent; adapted from Reed and Aspinwall [17]) × 2 (Integrated affirmation: texts present vs absent) factorial design testing two methods of affirmation (for a total of four conditions). Only 17 eligible users completed all *baseline affirmation* questions and these conditions did not influence any outcomes, so we collapsed across baseline affirmation conditions in analysis. Analytic comparisons were between standard intervention (consisting of the control condition and the baseline affirmation only condition) versus intervention enhanced with self-affirmation integrated throughout the 6-week program (consisting of the integrated affirmation only condition and the integrated plus baseline affirmation condition).

In the *integrated affirmation* conditions (hereafter referred to as “affirmations”), affirmations (which instructed users to focus on strengths or values when feeling threatened or anxious) replaced approximately 20% of texts delivering motivational content. On quit day and the 3 subsequent days, one text was randomly replaced each day. For all subsequent days, every 5th or 6th text was replaced to ensure that the remaining 11 affirmation texts were distributed equally (the last affirmation text was on day 38). The order of administration was constant for all users. Table 1 contains sample texts, created by JMT, WMPK, and RF based on the self-affirmation literature [18], and the motivational texts used in the control condition.

**Table 1:** Sample affirmation and control texts.

Day text was delivered	Affirmation message content	Control (motivational) message content
Quit day	When you feel like you might relapse, focus on your good experiences. Think about a time you made someone laugh.	Text your supporters & remind them of the big day. Make sure they have your back. We do! Text back CRAVE, MOOD, or SLIP for more support anytime.
Day 1	When you feel threatened by a craving to smoke, focus on your strengths. Think of a time you worked hard on something you care about.	You have good reasons for quitting. Say them out loud daily to help keep you on track, especially when you are feeling low.
Day 3	When you feel anxious about quitting smoking, focus on your values! Think of a time you showed compassion for another person, even if it was hard.	Stay away from people/places that make you think of smoking. You will find it easier to cope that way (and you will avoid secondhand smoke).
Day 12	Quitting is hard! When you feel a craving, think of a time you learned from a mistake.	Congratulations! Being smokefree means no longer cheating the things you love for something that doesn't love you back.
Day 24	When you feel threatened by a craving to smoke, focus on something important to you. Think of a time you helped a friend, even if you felt busy.	Strong healthy bones are another benefit of quitting. Quitting smoking reduces your risk of bone fractures now & later in life. Text STOP to end.

### Measures

We assessed the number of days users remained enrolled following their quit date. Users who did not disenroll were assigned a value of 42.

Point prevalence cessation was assessed on users’ quit date and weekly thereafter until day 42 with minor variations on, “Are you still smoke free? Reply: YES or NO.” The qualitative responses from texts were recoded into quantitative values and uninterpretable responses were coded as missing.

Smoking frequency at enrollment (everyday, most days, some days, less than that) was collapsed into everyday versus other responses.

### Overview of Analyses

We conducted analyses on: (1) those meeting eligibility criteria (initiators, *n*=1261), (2) those remaining enrolled throughout the 42-day intervention (completers, *n*=687), and (3) those reporting smoking status at 6 weeks (responders, *n*=81) (see Tables 2 and 3). These *a priori* criteria ensured that users could receive the baseline affirmation the night before their quit date, and the same criteria were applied across all conditions. Four outcomes were assessed: days enrolled, completion of intervention (dichotomous), smoking status at day 7 (1 week), and smoking status at day 42 (6 weeks). The former two outcomes were only assessed among initiators, as all completers and responders remained enrolled throughout the 42-day intervention. Among initiators and completers, nonresponse to smoking status was recoded as smoking (even if users disenrolled). SPSSv.21 was used to run *t*-tests and chi-square analyses testing for differences in the dependent variables as a function of condition.

**Table 2:** Attrition and response rates as a function of study condition.

	Affirmation *n*	Control *n*	Total *n*	Percentage of treatment initiators
Met eligibility criteria for analyses (treatment initiators)	650	611	1261	--
Remained enrolled through 42-day intervention period (treatment completers)	363	324	687	54.5
Responded to 42-day smoking cessation item (treatment responders)	40	41	81	6.4

**Table 3:** Reasons for ineligibility for analyses as a function of study condition.

	Affirmation *n*	Control *n*	Total *n*
Total number of users that were not eligible	856	942	1798
Under 18	0	13	13
Did not set a valid quit date (ie, did not set a quit date or set date for 3014)	37	62	99
Did not set a quit date at least one day after the day of enrollment and thus may have already tried to quit before receiving any affirmations^a^	613	669	1282
Did not remain enrolled in the study through selected quit date	172	183	355
Set quit date too far into the future	53	50	103

Note: Some users were ineligible for more than one reason

^a^Self-affirmation has been shown to be ineffective when introduced after defensive responses to a threat [19]; thus, individuals who did not have the opportunity to affirm prior to or at the time of trying to quit were ineligible.

## Results

Among initiators, 35.8% (452 of 1261) were male and mean age was 35.5 years (SD=11.9, range=19-79). Most users (91.75%; 1157 of 1261) smoked daily. Among initiators, there were no significant differences in gender, age, or baseline smoking frequency across conditions (all *P*s>.22). Completers (*t* (1231)=−4.22, *P*<.001) and responders (*t* (1231)=−2.49, *P*=.010) were on average older than non-completers and non-responders, respectively, but did not differ in gender or baseline smoking frequency.

Among initiators, self-affirmation did not significantly improve any of the following: (1) intervention completion (54.6% overall; control: 324 of 611; affirmation: 363 of 650; *χ*^2^(1)=1.01, *P*=.315), (2) days enrolled (control: *M*=26.8 days; SD=17.5; affirmation: *M*=27.4 days; SD=17.7; *t* (1259)=−0.59; *P*=.553), or (3) 6-week smoking status (6% had quit overall; control: 32 of 611; affirmation: 39 of 650; *χ*^2^(1)=0.35; *P*=.557). Initiators who received affirmations were somewhat *less* likely to report cessation (9%, 61 of 650) at the 1-week follow-up than those who did not receive affirmations (12%, 75 of 611; *χ*^2^(1)=2.73, *P*=.098), although this was not statistically significant. Among completers, there were no significant effects of affirmation on cessation (1-week follow-up: 14% had quit overall, 49 of 324 control, 44 of 363 affirmation; 6-week follow-up: 10% had quit overall, 32 of 324 control, 39 of 363 affirmation, *P*s>.25).

Importantly, among the 81 responders, those who received affirmations were more likely to report cessation at the 6–week follow-up (98%, 39 of 40) than those who did not receive affirmations (78%, 32 of 41, *χ*^2^(1)=7.08, *P*=.008). There were no significant effects among responders for cessation at the 1-week follow-up (*P*=.57).

Of note, analyses including users who set quit dates 1) on the day of enrollment and 2) before or on the day of enrollment (testing whether any potential benefits extended to a sample of respondents that may have already attempted quitting) revealed no significant effects, consistent with evidence that self-affirmation is most effective before people can exhibit defensive responses to threat [19].

## Discussion

This proof-of-concept study provides preliminary evidence that self-affirmation can be integrated into existing text-based cessation programs, as affirmations did not lead to adverse effects (ie, less engagement or lower rates of cessation). Among those who reported smoking status at the end of the intervention period (6.4% of eligible respondents), affirmations facilitated cessation. However, when using an intent-to-treat approach with all eligible users, self-affirmation did not improve outcomes. Although effects demonstrated in this pilot study were modest, incorporating self-affirmations into the program was nevertheless simple and did not increase user burden - suggesting that even a small observed benefit is likely to be cost-effective.

Affirmations *interspersed* into an mHealth intervention allow users multiple opportunities to engage with the content and may promote cessation, at least among individuals who remain engaged with the program. Most users did not complete the baseline affirmation questionnaire. Users may have perceived the questionnaire as irrelevant. Future research might integrate an affirmation questionnaire into the registration process and clarify its relevance to users. Taken as a whole, these results suggest that the effect of self-affirmations within “low-touch” mHealth interventions may be most useful in specific short-term therapeutic windows. More research is necessary to determine appropriate dosing and timing of affirmation messages, and to understand the potential usefulness of self-affirmation messages on clinically relevant behaviors (eg, retention, engagement, and behavior change).

Although the potential generalizability of user data from a population-mHealth cessation program is a strength, these data also had limitations. Data were collected within an intervention, rather than controlled research protocol conditions, and thus were limited by the parameters of the clinical service. In addition, it is typical to incentivize participation in research settings, and the lack of incentives undoubtedly contributed to low response rates. Response rates may have increased if contact was made beyond that provided by the clinical service. Last, the cessation measures in the protocol were less optimal than smoking assessments of the last 7 days, and were not confirmed biologically.

We integrated an evidence-based approach (self-affirmation) into a real-world intervention delivered at the population level, providing a “proof-of-concept” that brief, low-touch interventions may be integrated into a text messaging program with potential benefits, minimal disruption, and little cost. However, many questions remain regarding how self-affirmation and similar approaches can promote engagement in population interventions.
